# A rapid communication from the AAPM Task Group 201: Recommendations for the QA of external beam radiotherapy data transfer. AAPM TG 201: Quality assurance of external beam radiotherapy data transfer

**DOI:** 10.1120/jacmp.v12i1.3479

**Published:** 2010-12-04

**Authors:** R. Alfredo Siochi, Peter Balter, Charles D. Bloch, Lakshmi Santanam, Kurt Blodgett, Bruce H. Curran, Martijn Engelsman, Wenzheng Feng, Jim Mechalakos, Dan Pavord, Tom Simon, Steven Sutlief, X. Ronald Zhu

**Affiliations:** ^1^ Department of Radiation Oncology University of Iowa Hospitals and Clinics Iowa City IA USA; ^2^ UT MD Anderson Cancer Center Houston TX USA; ^3^ Department of Radiation Oncology Washington University Saint Louis MO USA; ^4^ Department of Radiation Oncology Allegheny General Hospital Pittsburgh PA USA; ^5^ Department of Radiation Oncology Rhode Island Hospital Providence RI USA; ^6^ Center for Proton Therapy Paul Scherrer Institute Villigen Switzerland; ^7^ Radiation Oncology New York Presbyterian Hospital New York NY USA; ^8^ Dept of Medical Physics Memorial Sloan‐Kettering Cancer Center New York NY USA; ^9^ Radiation Oncology Vassar Brothers Hospital Poughkeepsie NY USA; ^10^ Sun Nuclear Corporation Melbourne FL USA; ^11^ Radiation Therapy VA Medical Center ‐VA Puget Sound Health Care System Seattle WA USA; ^12^ Dept. of Radiation Physics ‐ Unit 1150 UT MD Anderson Cancer Center Houston TX USA

**Keywords:** quality assurance, record & verify, treatment planning, DICOM, data transfer

## Abstract

The transfer of radiation therapy data among the various subsystems required for external beam treatments is subject to error. Hence, the establishment and management of a data transfer quality assurance program is strongly recommended. It should cover the QA of data transfers of patient specific treatments, imaging data, manually handled data and historical treatment records. QA of the database state (logical consistency and information integrity) is also addressed to ensure that accurate data are transferred.

PACS numbers: 87.56.bd, 87.55.D, 87.55.Gh, 87.55.km, 87.55.Qr, 87.55.T

## I. INTRODUCTION

This rapid communication comes from a Task Group of the Working Group on Information Technology, TG 201: Quality Assurance of External Beam Treatment Data Transfer. Each author listed five to ten QA and safety related recommendations based on their experience with particular models of treatment planning systems (TPS), treatment management systems (TMS, typically a combination of an oncology information system with a verify and record system^(^ ^1^ ^)^, and external beam treatment units. The collective experience covers a broad range of manufacturers and combinations of systems: Varian (Eclipse, ARIA, VARiS, Clinacs) (Varian Medical Systems, Palo Alto, CA), Philips Pinnacle (Philips Healthcare, Andover, MA), Elekta (Multi‐Access, Lantis, Mosaiq, Xio, GammaKnife, linacs) (Elekta, Stockholm, Sweden), Siemens linacs (Siemens Medical Solutions, Malvern, PA), IBA (Belgium) and Hitachi (Tokyo, Japan) proton units, TomoTherapy treatment units (Hi·Art) (TomoTherapy Inc., Madison, WI), and CyberKnife (Accuray Inc., Sunnyvale, CA). The task group organized over 100 recommendations into more general ones that cover one or more of the original recommendations. The QA related recommendations are the subject of this report, while the remainder, covering product design and standards, will be addressed in a separate report. In the interim, manufacturers should read this document with an eye for product improvements that can improve overall safety.

This report does not give descriptions of the various systems and the exchange of data among them. It is assumed that medical physicists who wish to implement these recommendations understand the systems in their clinic. A full task group report with more details is in progress and will be released at a later date. The purpose of this rapid communication is to provide clinics with a checklist and a diagnostic tool that can help determine what data transfer related quality assurance steps need to be implemented to make their radiation treatments safer. While some recommendations do not directly deal with the QA process, they do relate to our ability to perform QA and improve the safety aspects of treatment data transfer. Clinics should also consider implementing the necessary infrastructure described in many of the recommendations.

The first section of the report covers administrative issues in the clinic and focuses on the management of a QA program and clinical workflows. This is followed by a section on the treatment data that describes patient specific QA, manually handled data and the historical treatment record. The state of the treatment database is examined next in terms of data integrity and logical consistency of related data. Finally, imaging issues for planning and verification are addressed.

## II. ADMINISTRATION

Like any other quality assurance program, data transfer QA needs to be implemented in a systematic and comprehensive way. It needs to be managed so that its impact (or failure) is established through metrics, and a mechanism is defined to improve it and adapt it to changes within the clinic. Roles and responsibilities of individual physicists and other personnel need to be defined to establish accountability.^(^ ^1^ ^)^

This section describes the basic recommendations that pertain to the overall establishment and maintenance of a data transfer QA program. In addition, data transfer depends critically on the clinical workflow, especially when the data involves human interaction (e.g., manual data entry). Therefore, the second part of this section gives recommendations specific to the clinical workflow.

### A. QA program

#### A.1 A data transfer QA program should be established by a medical physicist.

Although QA procedures will require input from all staff, medical physicists are the most qualified individuals to design the program.^(^ ^1^ ^)^ They understand the flow of data from imaging to treatment, and are responsible for ensuring that the delivered treatment matches the physician approved plan.

#### A.2 The QA procedures should test patient‐specific treatment data transfers from imaging devices to the TPS, from the TPS to the TMS, from the TMS to the treatment unit, from the treatment unit to the TMS, and from the treatment unit or TMS to the TPS.

Treatment parameters for a given plan should be compared across all systems, including paper documentation forms and charts that exchange these parameters. Data transfer QA complements measurements or independent calculations of dose distributions. It should be designed within the context of treatment unit QA. The results of such tests should be documented in the patient's chart so that they can be reviewed.

#### A.3 Clinical treatment scenarios should be used when verifying the automated transfer of treatment data from the planning system to the TMS via DICOM^(^ ^2^ ^)^ or other file formats (e.g., RTP Connect, RTP Exchange).^(^ ^3^ ^)^

The functionality of all applications^(^ ^4^ ^–^ ^7^ ^)^ involved in either the verification or the transfer should be tested using clinical scenarios to rule out any systematic errors such as erroneous coordinate conversion or labeling. The test patients should represent all scenarios treated in the clinic (e.g., electrons, wedges, positioner rotations, IMRT, IGRT). New scenarios should be tested as they arise.

#### A.4 QA of any system in the data transfer chain should be performed both periodically and whenever changes are made. Data exchange at the interfaces of a changed system should be tested with benchmark cases.

New or updated systems and procedures can change the behavior of data transfers. QA should be performed similarly to an acceptance test procedure or commissioning step, and new procedures should be validated with end‐to‐end tests. QA should also be performed periodically as determined by the medical physicist. For example, an MLC test pattern (e.g., a diamond shape) downloaded from the TMS to the treatment unit could be included as part of the daily warm‐up tests. The results of QA tests should be documented in the maintenance logs for the systems involved in the tests.

#### A.5 The QA program should be evaluated by using benchmark cases with known data transfer problems.

This provides a quantitative evaluation of how well the QA program catches errors due to data transfer or treatment planning, for example. Appropriate testing, through the use of induced errors, can help ensure that the QA program was properly designed. It can also help point out areas where modifications are needed. Benchmark cases, not real patients, should be used to avoid introducing errors into clinical treatments. Such cases could be created by purposefully changing information in data sources or destinations (e.g., using a DICOM editor to change header data, or changing treatment machine parameters on the TMS and determining if the QA catches the difference from the TPS parameters).

#### A.6 The QA program should be re‐evaluated and, if necessary, modified in response to information and experience shared amongst clinics, in order to proactively avoid problems.

Although national radiotherapy error databases are currently under development, clinics should seek out information on data transfer errors that other clinics have experienced, and work with vendors to ensure the timely receipt of customer technical bulletins and advisories. They should also find out what mitigations, if any, have been successfully implemented so that they can adopt these safeguards. They could also use systems like RASMAS (Risk and Safety Management Alert System)^(^ ^8^ ^)^ that gather product alerts and recalls from multiple sources and disseminate the information to subscribers.

### B. Clinical workflow

#### B.1 A robust clinical workflow that includes checkpoints at all data exchange interfaces should be established. The workflow should be updated when new systems or subsystems (hardware, software or procedures) are implemented.

Errors should be caught at the point where they originate (or as close as possible), and independent checks should be used. Errors will more likely be caught if multiple independent mechanisms are involved in the verification of the data. For example, a second MU calculation by a different physicist is one checkpoint between the TMS and the TPS.

Ideally, a given data transfer should be verified prior to the next step in the workflow. Note that these data transfers are not only electronic but also involve other communication modes (e.g., verbal and written instructions, worksheets) and these must be well‐defined to avoid miscommunication.

#### B.2 All clinical workflows should be documented, with all documents readily available to all participating staff.

Workflow procedures include information about the data transfers that occur in the system, including instructions that originate from human sources (e.g., physician instructions) and how the information should be captured to ensure it is properly communicated.

#### B.3 All personnel involved in the data transfer chain should receive training prior to their participation in data transfers.

Clinical processes often have assumptions that can only be met if procedures are followed, so training in applications and clinic in‐services are important. To make the processes more robust (e.g., designing constraints to avoid deviations from standard procedures), physicists or teams of physicists should collectively understand the data transfer mechanisms and their interactions with clinical processes well enough to know where they might fail or cause risky situations. All staff should be able to report unusual system behavior. Such training should be documented and competencies evaluated annually.

#### B.4 Users should test the DICOM^(^ ^2^ ^,^ ^9^ ^–^ ^11^ ^)^ compatibility of their systems both in the import and export steps as part of commissioning. Where incompatibilities exist, the user should create well‐documented processes to address them.

While the compatibility of all files involved in data transfers is important (see II.B.1), most new technology will be using DICOM. One can compare the data in source and destination systems (e.g., by simultaneous viewing of screen captures or print outs of images and/or treatment parameters). Items that do not transfer correctly and require other transformation steps can be documented as requiring special attention and procedures. For example, some manufacturers export DRRs without scaling information. In the case of DICOM‐RT‐ION,^(^ ^11^ ^)^ the standard continues to develop with the delivery technology and systems are subject to many incompatibilities.

#### B.5 Warning and error messages should not be ignored. Users should notify the physicist, investigate the messages and document their findings.

On some systems, warnings tend to be dismissed when too many of them are presented to the user in the same way. However, a culture of “click through the warning messages” should be discouraged. The error should be investigated and documented so that all users will know how to respond to each error. Manufacturers should make error messages clear, specific and helpful, including suggestions on how to resolve the error.

#### B.6 Items that are used in the TPS but that need to be manually entered or modified in the TMS should be included in a checklist to remind TPS users to complete the manual transfer.

Clinics should design their own “standard” checklists (this can also be in the form of in‐house applications^(^ ^6^ ^)^. For example, information about bolus is not propagated from Pinnacle to MOSAIQ, and the bolus thickness has to be entered in the treatment field and notes. Similarly, prepopulated fields with clinically reasonable values that are different from the physician's intent may be overlooked, and these default items should be part of the checklist. Examples include table positions and dose rates.

#### B.7 Clinics should have policies and procedures in place to handle treatments that are interrupted by network or software problems.

Network or software problems that occur in the middle of a patient treatment can cause the transfer of information to be interrupted, potentially causing delivered treatments to go unrecorded in the TMS, and patient treatments to be interrupted. Users should have procedures and policies in place to deliver and record the remainder of the treatment or, if this is not possible, to plan a course of action to manage the rest of the patient's treatment course. This also applies in the case of a power outage.

Recovery procedures will consist of the following general steps: 1) determine the state of the treatment delivery unit at the time of the interruption, 2) assess what part of the treatment was delivered, 3) attempt to deliver the remainder, and 4) reconcile the information in the TMS by manually recording parts of the treatment that were delivered but not recorded. Refer to the manufacturer's instructions and seek help, if needed, to accomplish these tasks. If it is not possible to deliver the remainder, the physician should decide how to change the patient's treatment schedule.

#### B.8 Clinics should adopt a change driven QA paradigm and check the TMS when activities with the potential to change treatment data occur.

If the prescription is changed after a plan is entered, an independent review should be done to ensure the plan is still appropriate. A simple change, such as increasing the number of fractions, could cause critical structure tolerance doses to be exceeded. Other examples include bolus changes, reshaping electron cut‐outs, and changing patient setups for clearance. A more serious example might require a new plan on a different treatment unit with different energies due to the current unit being down. Such “activities” should trigger formally established procedures so that there is clear communication among all those involved and the appropriate actions are taken.

## III. TREATMENT DATA

In addition to establishing a QA program and managing clinical processes, there are recommendations specific to patient treatment data. These recommendations fall into three categories: pretreatment patient‐specific QA, manually entered treatment data, and historical treatment records.

### A. Patient‐specific QA

#### A.1 Whenever possible, patient‐specific QA of data transfers should be implemented on the actual data that will be used for treatment, rather than a copy of the data.

One should verify whether a copy is used (e.g., earlier versions of VARiS or ARIA can create QA fields) or the actual fields are treated without recording dose for the patient (QA mode on later versions of ACCESS, MOSAIQ and ARIA). Unless the copy is compared to the original to ensure they are exactly the same, tests on the copy will only give you confidence to treat with the copy. The records that will be downloaded for the actual patient treatment could be different.

#### A.2 Clinics should perform patient‐specific verification of treatment parameters in the treatment database to ensure that they match those in the treatment plan, prior to approval for treatment. This includes all control points in a delivery sequence.

Certain methods of patient‐specific QA have been used as surrogates for comparing control points, such as IMRT QA^(^ ^12^ ^)^ and the comparison of the Complete Irradiated Area Outline (CIAO). While these may catch gross errors and prevent accidents, they may not pick up subtle variations in treatment parameters that have the potential to compromise treatment quality. Checking a representative shape for a DMLC plan (e.g., CIAO) does not guarantee that the control points are correct. Hence, a control‐point‐by‐control‐point comparison is needed. This can be done with in‐house applications^(^ ^6^ ^)^ or subsystems of commercial applications (e.g., graphical comparisons, dose map or fluence profile comparisons). These should all be done prior to approval for treatment, since the large number of MUs involved in IMRT could cause a lethal over dose within a couple of fractions if DMLC files are missing.^(^ ^13^ ^)^

While such a comparison represents an ideal, there may be logistics issues. Clinics should find reasonable methods to approach the ideal, and manufacturers should provide and incorporate tools to ensure database integrity (see Section IV) and perform exact comparisons of data on both sides of the transfer.

#### A.3 The transfer of coordinate system‐dependent data (images, dose, and treatment parameters) should be verified for proper orientation and registration.

Flipped orientations from left to right have been noted in imported images for some systems. Flipped orientations from superior to inferior have been observed in exported dose data from other systems. If treatment plans or image guidance shifts are based on this information, this could lead to treating healthy normal tissues while missing the tumor. Particular attention needs to be paid to nonstandard treatment geometries such as prone and/or feet‐first, and such functionality should be tested prior to first use. To avoid potential left/right confusion, methods such as asymmetric placement of fiducials should be considered and are highly recommended for cases without visible anatomical changes.

#### A.4 Independent MU checks should be performed on the data that gets downloaded to the treatment unit.

The detectability of data transfer errors can be increased if an MU check is performed on the TMS data rather than the TPS data. Recommendations for MU check procedures will be described by TG‐114, while MU checks for IMRT are described in the literature.^(^ ^6^ ^,^ ^14^ ^)^

#### A.5 For RT systems that involve the transfer of treatment planning data directly to the treatment delivery unit (automatically or manually), procedures should be in place to verify that the delivery data match the planning data.

Some models of some systems require data to be programmed at the treatment unit. An independent check should verify that the programmed data were correctly entered or transferred.

### B. Manually‐handled data

#### B.1 Items that are manually entered into TMS or imaging systems should be checked to ensure they match the corresponding items in the treatment plan or in the patient's records (e.g., physician prescriptions).

Data transfers from the TPS to the TMS may be missing information that has to be entered manually into the TMS (e.g., number of treatment sessions per week, or per day, session or daily dose limits, field names, tolerance tables, setup instructions and verification image acquisition parameters). The manual entries must be checked by someone other than the person who entered them. The information is not always found in the planning system, and entries in the patient's chart must be consulted after verifying that these match the physician's intent.

#### B.2 Items that are manually positioned for treatment delivery should be checked to ensure they match the correct patient, site and field.

Some blocks, bolus, wedges, BrainLAB Topo alignment box overlay, and field‐specific hardware for protons (compensators, snout) cannot be verified by a TMS. Some type of interlock mechanism or tagging system (e.g., barcodes) may be needed to ensure the correct items are used for a given patient, treatment site and field. In the absence of interlocks, someone other than the person who placed these items should perform a verification of the item and placement.

#### B.3 For radiotherapy systems that are not directly tied into an electronic medical record or TMS, procedures should be in place to ensure that the appropriate information is recorded in the patient's chart.

Gamma Knife systems, for example, have minimal support for TMS (billing and scheduling only). For plan or treatment information to be included in the chart, pdf files are imported into the TMS or electronic medical record. Similar situations arise for some versions of other systems, such as the CyberKnife and TomoTherapy.

#### B.4 Mechanisms that transfer clinical setup data (e.g., simulator, virtual simulation) to the TMS should be checked.

When a TPS is not used to generate a plan, other methods that export plan data should be verified. For example, some simulators capture plan settings and export them to the TMS. Items that are not yet known but are required for import (e.g., energy, MUs) should be verified to ensure that default values are not erroneously accepted. These plans should all be independently checked prior to treatment, with particular attention to the dummy values.

In addition, improved visualization of patient position from outside the room would reduce the occurrence of errors arising from different coordinate system conventions (e.g., simulator gantry angles that are offset by 180° from linac gantry angles). When all parameters can not be verified remotely, users should enter the room to verify all positions.

#### B.5 Amendments to a treatment plan should be recorded in the TMS or TPS, and be independently verified.

Treatment plans sometimes require modification to handle items that cannot be easily taken into account. For example, couch attenuation will require more MUs, and changes in compensator thickness modify proton therapy ranges. If the TPS is unable to model the situation, it could be included as an amendment record or documented in the TMS or TPS. A physicist or dosimetrist other than the one who added the amendment should verify the data.

### C. Historical treatment record

#### C.1 Dose tracking problems should be resolved prior to the next treatment delivery to ensure the proper operation of dose‐based system functions.

The dose for a partially treated fraction may not be recorded correctly by the TMS (e.g., treatment unit breaks midway through a treatment). This could lead to a deviation from the physician's intent over the treatment course. Procedures must be in place to ensure that appropriate doses, prescriptions and dose action points are modified so that instructions related to the patient's receiving a particular cumulative dose can be carried out at the right time.

#### C.2 TMS users should devise procedures to correctly track dose for situations that the TMS can not handle.

For (passively scattered) proton therapy, the decision on which fields to deliver may change at each fraction. While a plan may consist of many fields, a fraction may be a subset of the fields. This is also true for other situations (total skin electrons, bolus every other day). Many dose tracking schemes do not accommodate these techniques and thus the probability of errors is increased. Treatment calendars should be reviewed as their incorrect use may also introduce dose tracking errors.

#### C.3 Delivered treatments should be compared against the intended plan to ensure complete accuracy.

One suggestion is to use portal dosimetry during patient treatment. Another is to augment the weekly chart check (i.e., reviews of the TMS treatment history log) by searching for delivery parameters (including DMLC control points) that are out of tolerance. Such methods are still under development by various academic institutions and manufacturers should take advantage of this work to implement the solutions commercially.

#### C.4 All treatment plans, including modified versions of existing plans, should be checked prior to treatment.

A combination of the treatment record and corresponding plan dose distributions will be required to create the composite dose distribution a patient received. Checking plans prior to treatment will improve the accuracy of this dose determination. If the patient is to receive later courses, it is important to know the patient's dose history.

Users should document changes in the planned treatment with the treatment plan so that the required information is in one place. The electronic treatment record can be used as verification, if necessary. The correct labels and plan versions should be verified. Arbitrary and undocumented changes to a treatment (e.g., collimator and gantry rotations) should be forbidden and should force a new plan to be calculated and verified prior to treatment.

## IV. DATABASE STATE

While the main focus of this document is on the QA of data transfer, it is meaningless if the source data are corrupt. For this reason, the patient database must have routine QA to ensure its integrity. In the broad scope, we have made recommendations to assure the accuracy of the data transfer from the treatment planning system to the patient database. Further recommendations were made to ensure this data is accurately transferred to the treatment machine. However, even if these transfers are error free, the data at the treatment machine will not match the treatment planning data if the database became corrupted or modifications were made during the interim period between these transfers. Considering the additional transfer of actual treatment records from the treatment machine back to the patient database and the duration of a course of radiation therapy, it is absolutely necessary to ensure the database integrity is maintained.

### A. Logical consistency

#### A.1 Users should check all related data in the TMS and TPS for logical consistency. Inconsistent items should be corrected.

Some items can only be entered in the TMS (e.g., prescriptions) because they are not transferred or accurately captured in the TPS. This can lead to inconsistent states when the rest of the plan is exported to the TMS. Other examples include DRRs (the image may be put into the wrong field) and data propagated from the simulator. Checking for consistency among the data within the TPS and the TMS will reduce errors caused by conflicting information.

#### A.2 When checking a plan, physicists should check the TPS and the TMS for unusual data or departures from the norm.

Including a check for the absence of an MLC for an IMRT treatment could reduce the likelihood of accidents like the one reported in the NY Times.^(^ ^13^ ^)^ Also, physicists should be familiar with typical monitor unit settings and beam arrangements in order to catch deviations from standard practice.

#### A.3 Verify that a treatment unit is compatible with the parameters in the treatment delivery database. For example, compatible, beam‐matched machines can be used for treatment, and incompatible machines are forbidden.

Some treatment systems allow you to reset the values that do not match (e.g., if a given treatment unit does not have the right energy, one could be prompted to select an energy and complete the setup for missing parameters manually). This can cause a serious deviation from the intended dose distribution. On the other hand, an identical machine in every respect except for the name should be allowed to treat a patient in case the original machine malfunctions.

### B. Information integrity

#### B.1 When unintended changes to the treatment database are discovered, this should be followed by a comparison of the affected data against the treatment plan prior to the next treatment of the field. Periodic TMS database integrity tests can find such changes.

Scenarios exist where the treatment database and its supporting files can be inadvertently changed (e.g., unintended unapproval of treatment fields during a weekly chart check, windows directories being re‐arranged, primary database fails and is not synchronized with the backup). While the changes can be undone, there is no certainty that the database state has been completely restored. Some type of integrity test can restore that confidence. This can consist of automated checksums of all records in the database, and should be done for all patients under treatment prior to their next treatment fraction. One possible user test involves exporting each patient's treatment parameter file for a known good state (e.g., after approving the fields), and generating a message digest 5 (MD5)^(^ ^15^ ^)^ checksum of this file as a reference. The user can export the file as needed and calculate its MD5 checksum for comparison to the reference value.

#### B.2 Systems involved in the transfer of treatment data should be configured to prevent security risks^(^ ^1^ ^)^ (privacy, viruses, attacks) without compromising the treatment unit's ability to treat correctly and efficiently.

Treatment data for systems without anti‐virus software could be corrupted. All systems should have virus prevention policies. Operating systems prone to virus attacks (e.g., Microsoft Windows) should allow and/or require the end user to maintain up‐to‐date virus protection. For treatment units this virus mitigation should run in low priority mode, not real time, to prevent interference with treatments. (Auto‐run on Windows‐based machines should be turned off.) Users should work with manufacturers to resolve conflicts related to policies for the installation of such software. An alternative is to use a medical device subnet with a firewall.

#### B.3 For radiotherapy systems that use a single database for planning and treatment delivery, clinics should ensure that the intended treatment plan and the actual delivery data are synchronized.

If errors or unusual system behavior occur when the TPS saves information to the database, in theory the information displayed by the plan and downloaded to the treatment unit should still be exactly the same, since the TPS and the treatment unit share the same database. However, there is still an uncertainty as to whether they are displaying the same information, since they may be displaying information from separate, temporary local data stores. The display states of the TPS and treatment database should be synchronized to the contents of the database, allowing gross errors to be caught by inspection. This may simply involve restarting the affected applications or rebooting the TPS and TMS. A screen capture of the last modified or “crash‐affected” item on the TPS can then be compared to the corresponding item on the TPS to verify the synchronization.

#### B.4 Users of systems that do not rely on an external TMS (i.e., a TMS that is independent of the treatment unit) should check the integrity of the internal treatment database against the displayed plan and delivered treatment.

Traditional checks with a TMS allow the user to look at the TMS data and compare it against the settings on the treatment unit (e.g., accelerator jaw settings for a 10 by 10 field will have X and Y field sizes equal to 10 displayed on the TMS, the linac console, the field light and perhaps the hand pendant). A system without a TMS may have internal (or integrated) TMS functions (e.g., storing machine parameters such as leaf positions as a function of MU), but these may not be apparent to the user. The user will not be able to inspect the values of the internal database and verify that the machine was set correctly. Some suggested workarounds involve comparison of screen shots of the initial plan against screenshots of the plan downloaded for treatment, or MATLAB scripts (The MathWorks, Natick, MA) that take the delivered QA data and compare it to the delivered patient data. For example, TomoTherapy users will have to compare the delivered QA sinogram against the delivered patient sinogram.

## V. IMAGING

The final component of radiation therapy data QA relates to imaging. Image‐guided radiation therapy is becoming increasingly commonplace, and so image data becomes as critical as numerical beam data. The image data recommendations are divided into planning and verification data.

### A. Planning

#### A.1 Integrity of images transferred from imaging systems to planning systems should be checked. This includes image quality and patient demographics (e.g., name, ID).

When images are transferred from outside departments or clinics, they may undergo multiple transformations before they can be imported into the TPS. This can cause changes not only to the images (e.g., bit depth) but also to demographic information if they are entered multiple times. One must check that these images belong to the correct patient and are brought into the correct plan in the correct orientation.

#### A.2 The assignment of primary and secondary images for planning should be checked, specifically at the image registration stage.

Patients can have multiple studies and series within a study. Choosing the wrong study can provide useless registration information (e.g., a T2 MR was fused with the CT scan but the tumor was only visible in the T1 MR which was not used for planning). It can also cause problems with heterogeneity corrections (the CT used for the primary set does not correspond to the HU to density calibration table) and dose distributions.

### B. Verification

#### B.1 The transfer of image‐guided radiation therapy (IGRT) data from the planning system to the treatment unit's IGRT system should be verified to ensure the correct points of interest are matched to the correct treatment sites, and that reference and treatment images are registered.

The following errors are possible when IGRT data are not verified: a) a point other than the intended isocenter (e.g., a dose calculation point) is chosen as isocenter,^(^ ^16^ ^)^ b) multiple treatment isocenters cause confusion and the wrong treatment fields are associated with each isocenter, c) the coordinate system of contours may be different from those of the images, leading to a misregistration, and d) the coordinate systems of different elements in the data transfer chain may be different, leading to a patient position at the treatment unit that is different from the intended one. These can all lead to treating the wrong location. The following steps can assist in the avoidance of such errors: isocenters can be indicated on the reference images; reference images in the TMS can be compared to those in the treatment plan; only one isocenter could be treated per session; and indications of isocenters (e.g., crosshairs and text overlays of the coordinates) in DRRs and in 3D reference images can be compared to those in the plan visually and numerically. These concepts also apply to dynamic positioning systems such as the CyberKnife, where DRRs are used for tracking the target and for turning off the beam when tolerances are exceeded.

#### B.2 The transfer of imaging data from the treatment planning system to the TMS should be verified to ensure that the TMS and TPS display all images correctly.

Displays of verification images on the TMS should match the TPS, including MLC leaf position overlays on DRRs. Subtle display issues dealing with resolution can change scaling and distort the image and/or its associated overlays (e.g., graticules). Even if the TPS and TMS match, they may not display the intended information, and this needs to be verified by the physicist.

It is also possible that the transfer of data from two separate sources can leave the database in an uncertain state. (e.g., as of this writing, manual entry of treatments in MOSAIQ can cause images sent from the on‐board imager of the 4D integrated treatment console to be lost.) It is important to verify that all data have been correctly transferred.

## VI. CONCLUSIONS

As the complexity of radiotherapy increases, clinics must modify their QA program accordingly, with a clinical team lead by a medical physicist. While this traditionally meant an improved understanding of hardware, QA now requires greater knowledge of information technology and standards for data exchange (e.g., DICOM‐RT). Because data transfer is tightly coupled to clinical processes, a QA program must involve an analysis of clinical workflows, and will require additional physicists and IT staff. The QA program will enable physicists to develop appropriate patient specific QA procedures, as well as tests for manually handled data and methods for verifying the accuracy of the historical treatment record. An “under‐the‐hood” knowledge of the specific systems in the clinic will enable physicists to test the treatment database integrity and assess whether related data are logically consistent with each other. The transfer of images and their associated objects for planning and treatment verification must also be part of any QA program. Finally, the transfer of data has two aspects – the physical transmission and the communication of information. The recommendations in this report are made to support physicists in designing QA programs that test the integrity of the meaning behind the data (i.e., the information) so that our radiation therapy treatment deliveries are exactly what the physician prescribed.
